# Prehabilitation interventions to support the postoperative recovery of adult kidney transplant candidates: a scoping review

**DOI:** 10.1186/s12882-026-04759-7

**Published:** 2026-01-22

**Authors:** Jasjeet Chhoker, Jada Drennan, Janine Farragher

**Affiliations:** Department of Occupational Science and Occupational Therapy, Temerty Faculty of Medicine, University of Toronto, 160-500 University Ave, Toronto, ON M5G 1V7 Canada

**Keywords:** Chronic kidney disease, Kidney transplant, Prehabilitation, Rehabilitation, Exercise, Postoperative recovery

## Abstract

**Background:**

Kidney transplantation typically offers superior health outcomes than other renal replacement therapies for people with kidney failure. However, kidney transplant recipients still often experience residual complications of kidney failure, such as frailty, cognitive challenges, and/or anxiety after transplant. Prehabilitation has been proposed to improve post-transplant outcomes, but it is unclear to what extent prehabilitation interventions have been studied to support postoperative recovery in adult kidney transplant candidates (KTC).

**Methods:**

This scoping review followed the Joanna Briggs Institute methodology. Five electronic databases (MEDLINE, EMBASE, PsycINFO, Cochrane Central Register of Controlled Trials, and CINAHL Plus) were searched. All articles reporting on prehabilitation interventions (i.e. preoperative interventions providing exercise, nutritional, cognitive, psychosocial, and/or educational support) for KTCs were included. Title and abstract screening were divided and conducted independently by two researchers after initial inter-rater calibration, then full text screening and data extraction were performed in duplicate. Data analysis was completed using descriptive statistics and narrative synthesis.

**Results:**

Nine studies were found to be eligible and included in the review. Prehabilitation interventions described in the literature consisted of psychosocial (*n* = 3; 33%), physical training (*n* = 3; 33%), diet/nutrition (*n* = 1; 11%), educational (*n* = 1; 11%), and multi-domain (*n* = 1; 11%) interventions. Interventions were primarily delivered individually in a face-to-face format (*n* = 4; 44%) or a virtual asynchronous format (*n* = 2; 22%), and were typically delivered in outpatient clinics (*n* = 2; 20%). Eight categories of outcomes were assessed across the nine studies, which included cognition-based outcomes (*n* = 6; 67%), individual outcomes (*n* = 5; 56%), intervention specific outcomes (*n* = 4; 44%), and functional and/or performance outcomes (*n* = 3; 33%).

**Conclusions:**

This scoping review highlights a lack of research into prehabilitation interventions for KTC. It suggests a need for further research to support the development of comprehensive prehabilitation protocols for KTC, and more research into their functional impacts.

## Background

Approximately four million Canadians are affected by chronic kidney disease (CKD) [1]. CKD is a gradual loss of kidney function over time which can progress to end stage kidney disease (ESKD), a condition that affects about 48,375 Canadians [2]. While there is no cure for kidney failure, renal replacement therapies (RRT) including dialysis and transplantation are available to prolong life and improve symptoms [3]. Transplantation, while less common than dialysis, is a superior option as it generally offers fewer restrictions on everyday life, a greater quality of life, and a longer life expectancy compared to dialysis patients [4, 5]. Shi et al. [6] found that after 5 years, the survival rate for kidney transplant recipients (KTR) was 80%, whereas for dialysis the survival rate was 53%. Additionally, transplantation is more cost effective for the health care system and the user, as dialysis patients incur expenses related to travel costs, training, and increased water, electricity, and garbage disposal fees from home dialysis [7, 8].

While transplantation is widely agreed to be the preferred treatment modality for eligible adult kidney transplant candidates (KTC), it is not without limitations. Kidney transplant surgeries cause physiological stress and can lead to postoperative physiological difficulties including discomfort, pain, infection, and fatigue [9]. Recipients may also experience residual complications from kidney failure or dialysis, even after transplantation [10]. This can include co-morbidities, poor respiratory muscle strength, or vascular dementia, which is a cognitive impairment resulting from reduced blood flow to the brain [10–12]. Additionally, approximately 21% of patients with moderate to severe CKD experience frailty, defined as physiological decline and increased vulnerability to stressors [13]. While frailty is more common amongst older people with kidney failure, it can occur in all age groups [14]. Frailty can greatly hinder recovery after a transplant and is indicated to lead to delirium, graft rejection, greater complications after surgery, and mortality [15–17]. Psychological difficulties are also common amongst KTR, as they may face guilt or grief for their donor, anxiety of transplant rejection, or depressive episodes upon realizing the transplant will not restore their lifestyle to the way it was before kidney failure [18, 19]. These psychological factors may impact adherence to post- transplant medications, which may increase the risk of transplantation rejection [18]. The aforementioned physiological and psychological difficulties following organ transplant have been reported to limit daily activities and reduce quality of life [9]. KTR have described how postoperative treatment burdens create role and social activity limitations, and difficulties adapting to their new lifestyle following transplant [9, 20]. For example, fatigue prevents a large proportion of KTR from returning to work, a meaningful role that is important for many individuals’ quality of life [21, 22]. Overall, these post-transplant physiological and psychosocial difficulties can influence the postoperative recovery of KTR.

Given the prevalence of postoperative complications in KTR, it becomes necessary to develop strategies to prevent or reduce this impact. The preoperative period is suggested to be a better time to optimize patients’ physical and psychological status because they are usually in better conditions compared to the acute postoperative period [23]. Additionally, a growing body of literature has demonstrated that an individual’s health status prior to surgery predicts postoperative complications and functional, psychosocial, and surgery-related outcome [24]. However, while waiting for transplantation, KTC face many physical, mental, and social challenges which may limit their preoperative health status. The median wait time for adult KTC with kidney failure to receive a deceased donor kidney is about 3.5 years, during which time patients are often receiving dialysis [2]. As previously indicated, dialysis may cause physical and cognitive symptoms such as nausea, decreased concentration, and debilitating muscle cramps, and is associated with a high prevalence of frailty [25]. Dialysis patients with kidney failure also experience psychological distress including symptoms of depression, anxiety, stress, and insomnia [26]. While awaiting kidney transplant, CKD patients may also progress to other comorbidities, like cardiovascular disease, diabetes, and musculoskeletal disorders, which contribute to disease progression, transplantation rejection, and reduced quality of life [27].

While the pretransplant period involves many health challenges, several risk factors have the potential to be modified prior to kidney transplant to improve surgical outcomes [28]. Preoperative interventions referred to as “prehabilitation” refer to interventions that aim to optimize the fitness, wellbeing, and functional capacity of surgical candidates during the period before surgery to prepare them for their operation and post-operative recovery [14]. These interventions can include exercise, dietary, cognitive, and/or psychosocial interventions [14]. In recent years, numerous studies on prehabilitation have been carried out in cancer, orthopedic, and cardiac surgeries [29, 30]. Systematic review and meta- analyses of various surgeries concluded that prehabilitation interventions can reduce the length of hospital stay, and can possibly improve postoperative pain and physical function, compared to standard care [14, 30, 31]. Within solid organ transplant patients specifically, prehabilitation interventions have been shown to be a effective and safe for improving functional and surgical outcomes [14]. Additionally, prehabilitation has been shown to reduce the need for postoperative rehabilitation [32]. To date, it is unclear what prehabilitation interventions have been trialed to support postoperative recovery in adult KTC. The objective of this scoping review was therefore to identify and describe the literature on prehabilitation interventions to support postoperative recovery in adult KTC.

## Methods

This scoping review followed the Joanna Briggs Institute (JBI) methodology for scoping reviews [33]. Additionally, this scoping review manuscript was written according to the Preferred Reporting Items for Systematic Reviews and Meta Analyses Extension for Scoping Reviews (PRISMA-ScR) reporting guidelines [34].

### Search strategy

Two researchers (JC and JD) developed a comprehensive search strategy in consultation with a University of Toronto librarian. Electronic databases searches were conducted using a combination of predefined keyword terms (e.g. Medical Subject Headings) and free-text terms representative of the target population (KTC) and concept (prehabilitation interventions). The databases searched included MEDLINE, EMBASE, PsycINFO, Cochrane Central Register of Controlled Trials, and CINAHL Plus. Backwards citation searching was conducted by searching the reference list of articles selected for full-text review and relevant review articles identified through the initial search. The most recent search was completed on October 16, 2024.

### Source of evidence selection

**Fig. 1:**
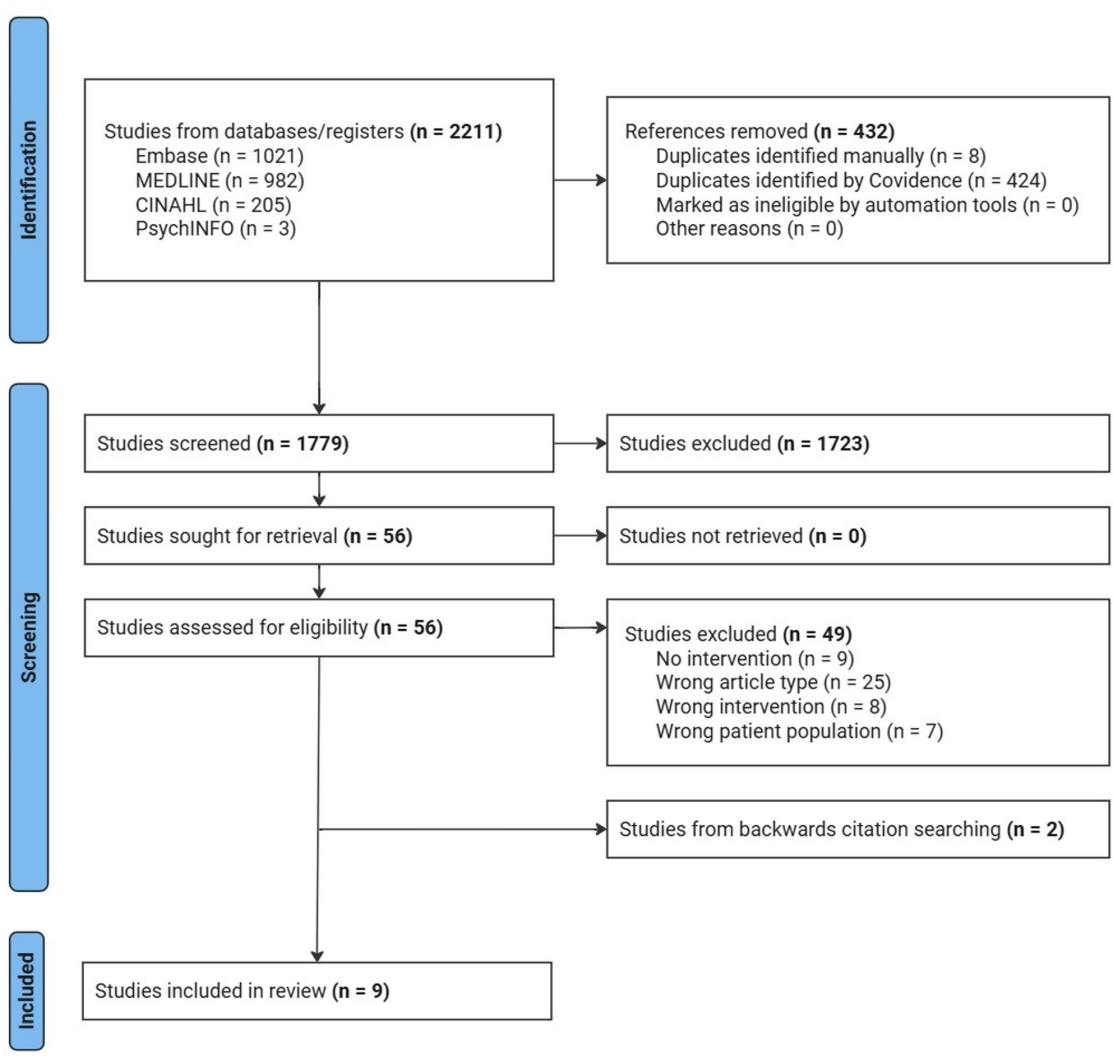
PRISMA-ScR flowchart of study selection process

All identified articles were imported to Covidence, a review management software, to remove duplicates and facilitate the screening process. For title and abstract screening, blinded pilot testing of the inclusion criteria was performed by the two independent reviewers (JC and JD) for a random sample of 180 articles in order to determine whether inclusion criteria were clear, appropriate and being interpreted consistently between reviewers [34]. Reviewers discussed conflicts and areas of improvement to study selection process as necessary, and screening began after over 85% agreement was achieved in pilot testing. Title and abstract screening were then divided and conducted independently by the two reviewers. Full text screening was conducted in duplicate, and any conflicts regarding inclusion of studies were resolved by discussion with a third reviewer (JF). See Fig. 1 for the study selection process [35].

### Inclusion criteria

Any qualitative, quantitative study or mixed-methods study describing the content and nature of a prehabilitation intervention used with the adult KTC population was eligible for inclusion, as per the inclusion and exclusion criteria presented in Table 1. KTC included any individual diagnosed with CKD or kidney failure waiting to undergo kidney transplant surgery, including those on dialysis. Prehabilitation was defined as any intervention implemented prior to a surgery that aims to enhance a patient’s overall physical and/or psychological function to support post-operative recovery. Prehabilitation interventions in eligible studies included, but were not limited to, physical exercise, nutritional support, psychological support, cognitive training, and educational programs focused on strengthening capacity and resilience for postoperative recovery. Preoperative educational interventions solely focused on educating KTC about the transplantation process and procedure were excluded. Studies meeting the above criteria, that were available in English from any year, or location, were included in the review.

**Table 1:** Study inclusion and exclusion criteria

Items	Inclusion Criteria	Exclusion Criteria
Participants	• Individuals over 18 years old diagnosed with chronic kidney disease and/or end stage kidney disease awaiting to undergo a kidney transplant surgery	• Individuals under 18 years old • Studies only about the kidney donor • Studies including organ transplant candidates other than kidney transplant
Concept	• Studies implementing and describing the content and nature of any prehabilitative intervention, defined as physical exercise/therapy, nutritional, psychosocial, cognitive, and/or educational interventions implemented prior to kidney transplant focused on promoting post-operative function. • Interventions must aim to enhance a patient’s overall physical, cognitive, and/or psychosocial health status.	• Studies describing only medical, pharmaceutical, and/or surgical interventions • Screenings, risk assessments and stress tests without further follow up • Education interventions solely focused on providing education about the transplantation process and procedure
Context	• Any setting, geographical location, or year of study	• Studies unavailable in English
Evidence Sources	• Full text primary research articles and reports • Quantitative, qualitative and mixed method designs	• Grey literature, study protocols, reviews, case studies, book chapters

### Data extraction and analysis

Data extraction was performed by two independent reviewers (JC and JD) in duplicate and stored in a secure Microsoft Excel sheet. Extracted data were discussed amongst the two reviewers to resolve discrepancies and reach consensus, and any disagreement were resolved by discussion with a third reviewer (JF). Data extracted included information on the study (e.g., title, authors, publication year, study design), population (e.g., age, sex, CKD stage), prehabilitation intervention (e.g., aims, duration, design), and study outcomes (e.g., outcome measures, reported findings). This process was guided by the Template for Intervention Description and Replication (TIDieR) to ensure comprehensive data extraction of interventions characteristics [36]. For the study interventions, the two reviewers (JC and JD) independently categorized each intervention into descriptive categories based on previously identified domains of prehabilitation, as described in Table 2. Similarly, outcomes were categorized according to outcome categories adapted from Grey et al. [37] (e.g., behaviours, cognitions, physiological measures, symptoms, health status, healthcare and other).

Data were analyzed using descriptive statistics and narrative synthesis. Counts and percentages were used to identify patterns in the literature, and narrative synthesis was employed to synthesize and describe the nature of prehabilitation interventions and their reported impacts on postoperative recovery in the KTC population. Consistent with scoping review methodology, we did not conduct a critical appraisal of individual studies, as our objective was to map the existing literature rather than evaluate study quality.

**Table 2:** Description of prehabilitation intervention domains

Domain	Description
Physical training	Physical exercise, movement, and functional mobility-based interventions and consultations with physical/respiratory therapists
Psychosocial	Psychological well-being and social support such as counseling, peer support groups, stress management techniques, or mindfulness training.
Cognitive	Remedial or compensatory strategies for supporting cognitive processing domains like executive function, memory, attention, etc.
Diet/Nutrition	Changes or guidance with diet and nutrition such as dietary modifications, nutritional supplementation, or nutritional counseling
Education	Providing supplementary knowledge and skills, such as self-management information, to strengthen post-operative functional outcomes
Multi-Domain	Combines two or more prehabilitation domains, delivered together as part of a single program.

## Results

A total of 2211 records were identified through database searches. After removing duplicates, screening and assessing articles for eligibility, a total of seven studies met the inclusion criteria for this scoping review. An additional two studies were identified from the backward citation searching of relevant review articles identified through the search. The PRISMA-ScR flowchart detailing the study selection process is shown in Fig. 1.

### Characteristics of included studies

A summary of the nine studies included in this review is provided in Table 3. Included studies were published between the years 1979 and 2022, with most studies being published in the last 10 years (*n* = 8; 89%). The studies were conducted in six countries, with the most common country of origin being the United States of America (*n* = 3; 33%). Study designs included randomized controlled trials (*n* = 3; 33%), quasi-experiments (*n* = 2; 22%), mixed methods (*n* = 2; 22%), pre-post (*n* = 1; 11%), and qualitative (*n* = 1; 11%). Sample sizes of the studies ranged from 21 to 286, with a median sample size of 52.

**Table 3:** Summary of prehabilitation intervention studies

Characteristic	No. of Studies (*n* = 10)
**Study design**	
Randomized Controlled Trail	3
Mixed-methods	2
Quasi-experiment	2
Pre-post	1
Qualitative	1
**Origin of study**	
United States	3
China	2
Germany	1
Hungary	1
Thailand	1
United Kingdom	1
**Year of publication**	
2019–2022	5
2015–2018	3
Prior	1
**Prehabilitation Setting**	
Outpatient clinic	2
Outpatient clinic & home-based	1
Inpatient hospital & outpatient clinic	1
Peri-operative hospital	1
Outpatient rehabilitation	1
Outpatient rehabilitation & home-based	1
Home-based	1
Not reported	1
**Provider**	
Multiple Providers	4
Self-administered	1
Respiratory Therapist	1
Psychologist	1
Certified MBSR Teacher	1
Nurse	1
**Delivery Format**	
Face to face (individual)	4
Virtual asynchronous (individual)	2
Face to face (individual & group)	1
Face to face and teleconference (group)	1
Face to face and self-guided (individual)	1

### Description and narrative synthesis of prehabilitation interventions

Table 3 summarizes the characteristics of prehabilitation interventions. Five categories of prehabilitation interventions domains were reported: psychosocial (*n* = 3; 33%), physical training (*n* = 3; 33%), diet/nutrition (*n* = 1; 11%), educational (*n* = 1; 11%), and multi-domain (*n* = 1; 11%) interventions (Table 4). Prehabilitation interventions typically took place in outpatient clinic settings (*n* = 2; 22%), with other settings including outpatient clinic & home-based (*n* = 1; 11%), inpatient hospital & outpatient clinic (*n* = 1; 11%), peri-operative hospital-based (*n* = 1; 11%), outpatient rehabilitation (*n* = 1; 11%), outpatient rehabilitation & home-based (*n* = 1; 11%) and home-based (*n* = 1; 11%). One study did not report the intervention setting (*n* = 1; 11%). Seven articles studied prehabilitation interventions exclusively, and two articles studied interventions which included both prehabilitation and non-prehabilitation components (*n* = 2; 22%).

**Table 4:**
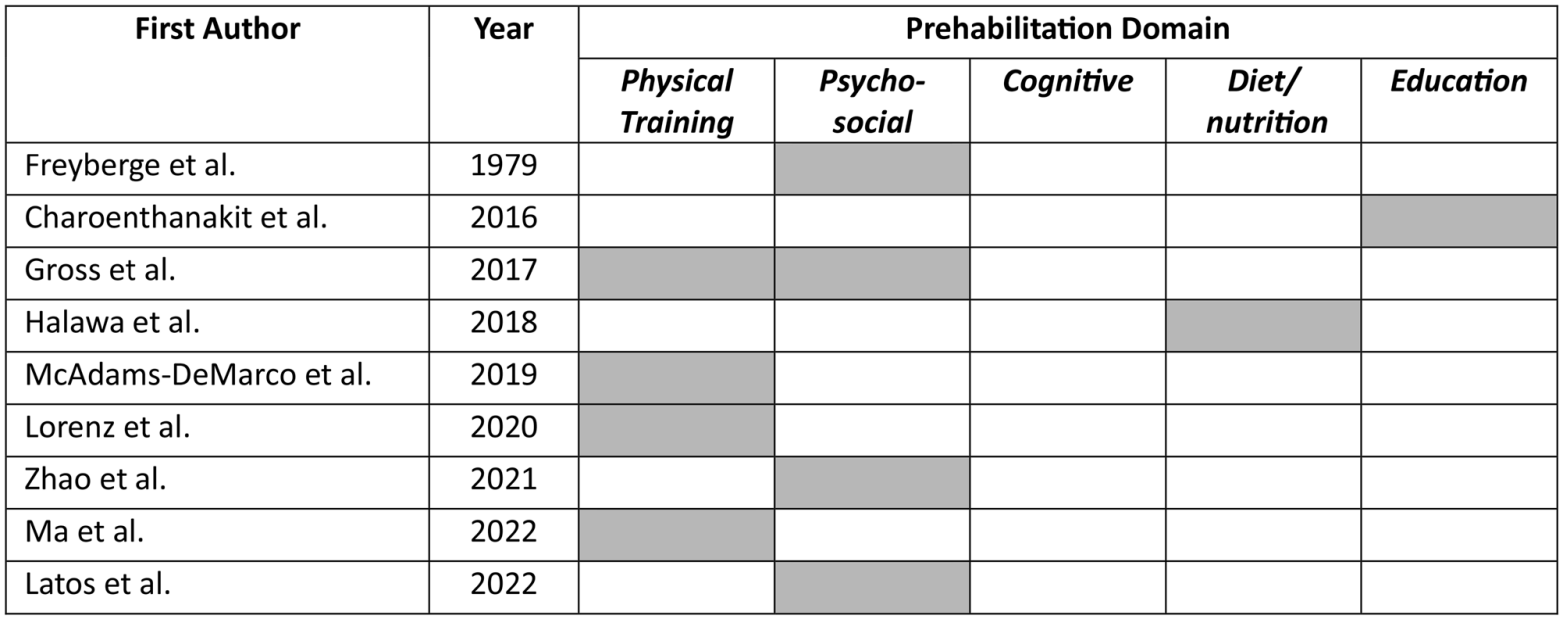
Prehabilitation domains addressed in included studies over time

### Physical training interventions

Physical training interventions were provided by physical therapists, physical therapy assistants, respiratory therapists (RT), and/or self-administered. Physical training interventions were delivered in an individual face-to-face (*n* = 1), individual face-to-face and self-guided (*n* = 1), and a virtual asynchronous (*n* = 1) format. Lorenz et al.’s [38] outpatient pulmonary rehabilitation (PR) program consisted of two ≤ 60 min sessions per week for 8 weeks with the RT. These sessions consisted of progressive, individualized PR exercises which trained endurance, strength, and flexibility. McAdams-DeMarco et al.’s [39] outpatient physical therapy (PT) and home program consisted of weekly 40-minute PT and/or PT assistant sessions with diaphragmatic breathing, stretching and strengthening, balance activities, range of motion activities, trampoline exercises, and cardiovascular exercises. Additionally, there were recommended daily at-home exercises between PT sessions. Ma et. al.’s [40] remotely supervised home exercise program consisted of a 12-week individualized exercise program including 40 min of aerobic exercise five times a week, functional resistance training three times a week, and 10 min of post exercise stretching. Exercises bracelets were given to participants, and information was uploaded to a WeChat group to track participation. Weekly phone calls from the experimenter were made to encourage patients to complete exercises.

### Psychosocial prehabilitation interventions

Psychosocial interventions were provided by nurses, psychologists, psychosomaticists, and/or student auxiliary therapists. All psychosocial interventions were delivered in a face-to-face format (*n* = 3) and were delivered in an individual format (*n* = 2) or combined individual and group format (*n* = 1). Freyberger et al.’s [41] consultation-liaison psychiatry program consisted of 1.5 group psychotherapy sessions every three weeks in addition to individual psychotherapy consultation and counseling. Latos et al.’s [42] 4-step positive psychology program included one 90-minute preoperative session once a participant was placed on the transplantation wait list. The session focused on empathy-oriented interventions to strengthen positive emotions. The remaining three sessions took place post- operatively, which are considered non-prehabilitation components of the intervention. Finally, Zhao et al.’s [43] psychological counseling program consisted of individualized psychological counseling where nurses analyzed individual participants’ psychological problems and adapted nursing accordingly.

### Diet/nutrition interventions

The diet/nutrtion intervention was provided by nurses and medical staff in an individual, face-to-face format. It consisted of carbohydrate loading through 2–8 high carbohydrate drinks spanning across the day of admission to the day of surgery. This program also included non-prehabilitation components including intraoperative fluid therapy and postoperative early mobilizations, laxatives, and medical pain management [44].

### Educational prehabilitation interventions

The education intervention was self-administered and delivered in a virtual asynchronous format, individually to participants [45]. It included a multimedia video-assisted instructional demonstration of scenarios to train patients in self-care practices [45].

### Multi-domain prehabilitation interventions

The multi-domain intervention consisted of psychosocial and physical training interventions provided by a certified MBSR teacher in a group face to face and teleconference format. It included medication, yoga and teacher-led group discussions [46].

### Description of study outcomes

Characteristics of the study outcomes are presented in Table 5. Outcomes fell into eight outcome categories including cognitions (*n* = 6; 67%), individual outcomes (*n* = 5; 56%), intervention specific (*n* = 4; 44%), functional and/or performance outcomes (*n* = 3; 33%), healthcare (*n* = 2; 22%), physiological measures (*n* = 2; 22%), behaviour (*n* = 2; 22%) and symptoms (*n* = 1; 11%). Certain intervention domains were more likely to report on specific outcome categories; for example, all psychosocial interventions reported cognition outcomes and all physical training interventions reported functional performance and individual outcomes. Overall, 48 individual outcome measures were used to assess the impacts of the prehabilitative interventions across the nine studies. Table 6 summarizes the details of the studies and their reported outcomes.

**Table 5:** Summary of study outcomes by category

Category	Description	Studies assessed	Studies reporting positive outcomes	Studies reporting mixed outcomes	Studies reporting negative outcomes
Cognitions	Changes in general kidney disease knowledge, self-efficacy, self-management, motivation, perceived stress, anxiety and fear, changes in cognitive domains.	6	3 (50%)	1 (17%)	2 (33%)
Individual outcomes	Quality of life, well-being and general satisfaction	5	3 (60%)	0 (0%)	2 (40%)
Intervention specific	Concepts regarding feasibility of intervention, enjoyment and interest in intervention	4	3 (75%)	1 (25%)	0 (0%)
Healthcare	Measurements of cost effectiveness, healthcare utilization and access	2	1 (50%)	1 (50%)	0 (0%)
Functional & Performance	Mobility measures, strength, frailty, endurance, energy levels, occupations (activities of daily living, leisure, productivity), return- to-work, assessments pertaining to everyday living	3	2 (66%)	1 (33%)	0 (0%)
Physiological measures	Changes in laboratory tests, blood pressure, vitals, body composition, and cardiovascular risk	2	1 (50%)	0 (0%)	1 (50%)
Behaviors	Adherence to diet, medication, physical activity, sleep, blood pressure control	2	1 (50%)	0 (0%)	1 (50%)
Symptoms	Changes in overall symptoms (i.e. pain, fatigue)	1	0 (0%)	0 (0%)	1 (100%)

Positive indicates significantly improved or qualitatively positive results. Negative indicates no significant change, significantly worse, or qualitatively worsened results. Mixed indicates individual measures describing outcome category included both positive and negative results.

**Table 6:**
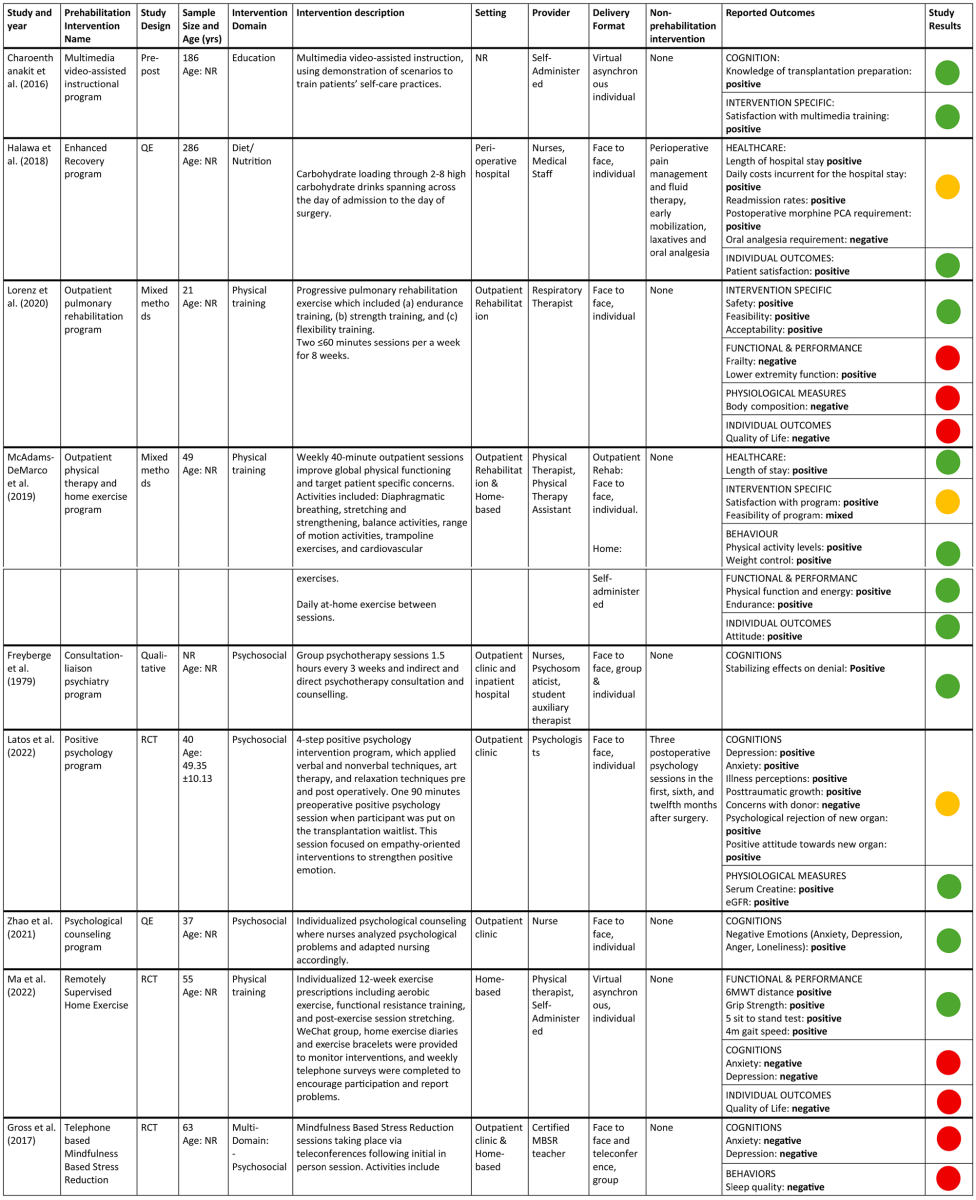

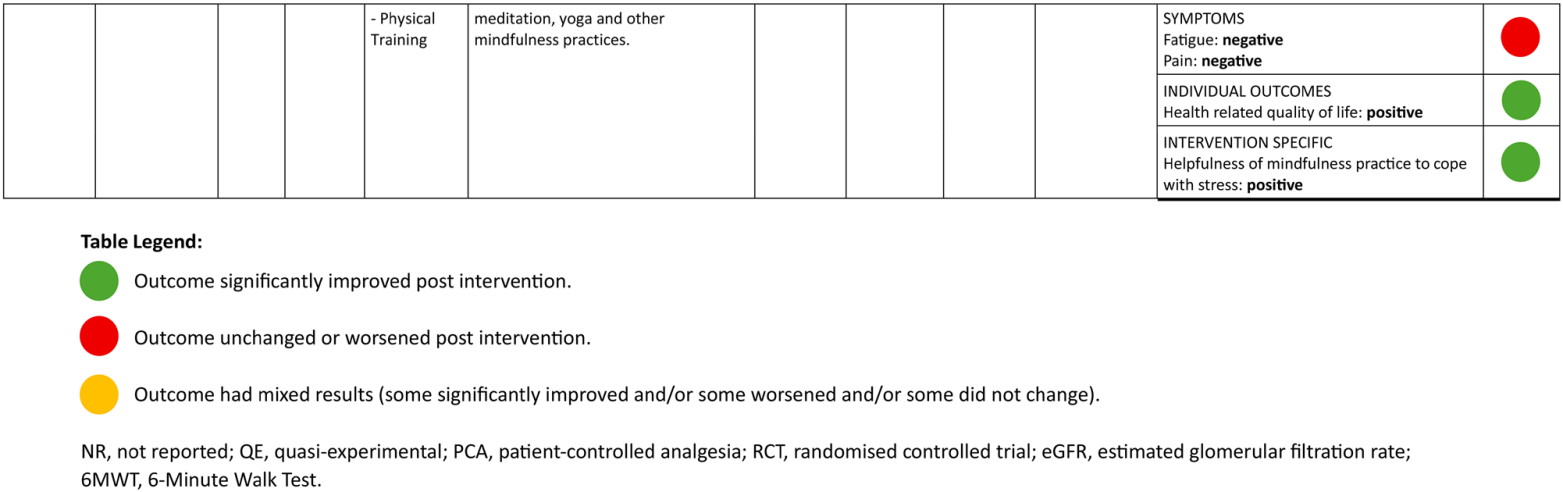
Summary of studies

## Discussion

In this scoping review, we sought to identify and describe prehabilitation interventions that have been used to support postoperative recovery in adult KTC. Our definition of prehabilitation interventions included exercise, dietary, cognitive, and/or psychosocial interventions aimed at optimizing the fitness, well-being, and functional capacity of kidney transplant candidates before surgery to prepare them for their operation and post-operative recovery [14]. We found that there has been a dearth of research on prehabilitation interventions in the adult KTC population, and that the majority of the studied interventions did not address the range of prehabiliative domains that KTC may benefit from to support optimal postoperative recovery. We also found that functional and performance outcomes were rarely assessed in the KT prehabilitation literature. The pre-transplant evaluation, preparation and waitlist period is an invaluable opportunity to introduce prehabilitation interventions to KTCs, as they typically have repeated clinical touchpoints with their transplant teams during this period [47]. This scoping review demonstrates the need to further investigate prehabilitation protocols that can support the functional recovery of adult KTCs, and to understand the impacts of such programs on patient-priority outcomes such as life participation and quality of life.

Our review revealed that only nine studies have investigated prehabilitation interventions for adult KTC, indicating a widespread dearth of research into this topic. A limited scope of research into prehabilitation has also been reported in other solid organ transplant populations [14, 48]; for example, Benmassaoud et al. [49] recently identified only six prospective studies or clinical trials focused on prehabilitation in liver transplant candidates. The limited literature in this field is incongruent with existing evidence indicating that prehabilitation can offer various benefits for solid organ transplant populations. For example, Quint et al.’s [14] review of studies on the effects of prehabilitation for kidney-, lung-, liver-, and heart transplant patient populations found prehabilitation to be an effective and safe intervention for improving the functional outcomes of solid organ transplant patients, and can lead to improvements in fatigue, frailty, cardiopulmonary fitness, anxiety, and overall quality of life. McIsaac et al. (50) systematic review found consistent evidence that prehabilitation based on exercise, nutrition, or multi-domain interventions that included exercise, may reduce rates of complication, improve health related quality of life, and improve physical status prior to major surgery. In addition to patient outcomes, prehabilitation can be economically beneficial in transplant populations by reducing postoperative complications and hospital stays [51–53]. There are several routine components of the pre-transplant pathway that could offer natural opportunities to incorporate prehabilitation interventions. For example, initial evaluation visits and mandatory education sessions could introduce basic physical activity guidance, fatigue management strategies, and/or psychosocial supports, while routine waitlist monitoring appointments provide opportunities to reinforce these skills and address emerging functional decline. For patients receiving in-center hemodialysis, intradialytic care also offers a stable setting to integrate simple strength or aerobic activities and/or self-management coaching. Embedding prehabilitation within these existing touchpoints may strengthen transplant readiness without requiring major changes to clinical workflows. As such, our findings support prior calls for increased research into this topic to identify the optimal components and modes of delivery of prehabilitation programs for KTCs [47].

Of the nine studies we identified in this review, we found that prehabilitation programs for KTC were largely focused on addressing a single domain of functioning. Only one study used a multi-domain intervention, despite KTC facing a myriad of challenges during the pre- and post-transplant period related to their physical and mental health. For example, frailty commonly occurs in KTR, which can lead to delirium and can increase the risk of mortality [13, 16, 17]. Anxiety of transplantation rejection is another common occurrence after a kidney transplant [19]. Multi-domain programs are recommended to be used instead of uni-domain approaches, as they address the complex interplay between physical and psychosocial factors [14]. For example, multi-domain interventions that include a physical exercise component have been suggested to be most likely to improve length of stay, quality of life and physical recovery metrics [50]. KTC have also indicated a preference for holistic interventions that address a range of their concerns [54, 55]. Multi-domain prehabilitation programs should therefore be prioritized [24, 47] to ensure KTCs receive support to overcome the diverse challenges they face in their recovery. To this end, we have identified multiple upcoming studies that plan to use a multi-domain approach to kidney transplant prehabilitation, suggesting there is growing attention being paid to this issue. For example, Perez-Saez et al.’s FRAILMar study [56] will consist of KTC participating in hour long exercises sessions 3 times a week for 8 weeks, in addition to psychological support throughout the intervention [56]. Patients will also be given protein supplements, and malnourished patients will be given an additional dietary supplement [56]. Quint et al.’s PreCareTx study [57] will be a 12-week program consisting of physical, nutritional, and/or psychosocial interventions depending on the patient’s need. Physical activity intensity, type, and duration will be individualized to the patient, and nutritional interventions will focus on optimizing energy, protein and other nutrients for individual patients [57]. In addition, psychosocial support will involve coping strategies, stress and energy management, social support, sleep hygiene, or relaxation techniques [57]. In addition to ensuring prehabilitation interventions address multiple domains of functioning, it will also be important moving forward to consider patients’ perspectives in the development of prehabilitation interventions [57], to ensure the resulting interventions meet patients’ needs and requirements.

Despite the ultimate goal of prehabilitation being to improve the functional capability of a patient prior to a surgical procedure [58], we found only three prehabilitation studies in the KTC population which measured functional or performance outcomes. Out of the 48 individual measures that were assessed across all studies included in this review, only 8 (17%) were functional and performance outcomes. Kidney failure and transplantation are known to have substantial impacts on function and daily life activities [9], and functional and performance status after kidney transplantation is related to quality of life. Similar to our scoping review, Ju et al. [59] identified life participation, described as engagement in meaningful activities of daily living, as a distinct construct in kidney transplantation that is infrequently assessed. However, life participation has been reported to be one of the most important patient-reported outcomes for kidney transplant patients based on consensus among patients, caregivers, and health professionals, indicating the need for its inclusion [60]. Functional status after kidney transplantation is also an independent predictor of post transplantation survival and mortality [61]. Functional assessments may help identify waitlisted patients who would benefit from prehab interventions to help survive transplant [61]. Based on this information, future studies should focus on assessing functional and performance outcomes when assessing the impacts of prehabilitation.

Our study has several strengths. We followed the JBI methodology for scoping reviews to ensure our review was comprehensive and met quality standards and followed the TIDieR checklist during data extraction to provide complete information about the interventions we identified [36]. While we were unable to screen article titles and abstracts fully in duplicate due to time resources, we conducted a thorough pilot of our initial screening protocol to calibrate our interpretation of the study inclusion criteria and ensure consistent screening across reviewers. Full text screening and data extraction were performed in duplicate to maximize the trustworthiness of our screening process. However, our study also had limitations. For example, we excluded studies that were not published in English, which might limit the generalizability of our findings to non-English speaking countries and regions. We also focused on published, full-text research articles to identify prehabilitation interventions for this review, which means that we might have missed prehabilitation interventions described only in grey literature.

## Conclusion

In conclusion, this scoping review has identified a lack of research into prehabilitation interventions for KTC, with current research featuring limited use of holistic interventions or functional outcome measures. These gaps demonstrate the need for further research into comprehensive prehabilitation protocols that can support the health and functioning of KTCs.

## Data Availability

No datasets were generated or analyzed during the current study.

## Abbreviations

CKD: Chronic kidney disease
ESKD: End stage kidney disease
JBI: Joanna Briggs Institute
KTC: Kidney transplant candidates
KTR: Kidney transplant recipients
MBSR: Mindfulness Based Stress Reduction
PR: Pulmonary rehabilitation
PRISMA: ScR-Preferred Reporting Items for Systematic Reviews and Meta-Analyses for Scoping Reviews
PT: Physical therapy
RT: Respiratory therapist
TIDieR: Template for Intervention Description and Replication
